# Therapeutic response assessment using 3D ultrasound for hepatic metastasis from colorectal cancer: Application of a personalized, 3D-printed tumor model using CT images

**DOI:** 10.1371/journal.pone.0182596

**Published:** 2017-08-10

**Authors:** Ye Ra Choi, Jung Hoon Kim, Sang Joon Park, Bo Yun Hur, Joon Koo Han

**Affiliations:** 1 Department of Radiology, Boramae Medical Center, Seoul, Korea; 2 Department of Radiology, Seoul National University Hospital, Seoul, Korea; 3 Institute of Radiation Medicine, Seoul National University College of Medicine, Seoul, Korea; 4 Cancer Research Institute, Seoul National University, Seoul, Korea; 5 Department of Radiology, National Cancer Center, Gyeonggi-do, Korea; Northwestern University Feinberg School of Medicine, UNITED STATES

## Abstract

**Background & aims:**

To evaluate accuracy and reliability of three-dimensional ultrasound (3D US) for response evaluation of hepatic metastasis from colorectal cancer (CRC) using a personalized 3D-printed tumor model.

**Methods:**

Twenty patients with liver metastasis from CRC who underwent baseline and after chemotherapy CT, were retrospectively included. Personalized 3D-printed tumor models using CT were fabricated. Two radiologists measured volume of each 3D printing model using 3D US. With CT as a reference, we compared difference between CT and US tumor volume. The response evaluation was based on Response Evaluation Criteria in Solid Tumors (RECIST) criteria.

**Results:**

3D US tumor volume showed no significant difference from CT volume (7.18 ± 5.44 mL, 8.31 ± 6.32 mL vs 7.42 ± 5.76 mL in CT, p>0.05). 3D US provided a high correlation coefficient with CT (r = 0.953, r = 0.97) as well as a high inter-observer intraclass correlation (0.978; 0.958–0.988). Regarding response, 3D US was in agreement with CT in 17 and 18 out of 20 patients for observer 1 and 2 with excellent agreement (κ = 0.961).

**Conclusions:**

3D US tumor volume using a personalized 3D-printed model is an accurate and reliable method for the response evaluation in comparison with CT tumor volume.

## Introduction

Colorectal carcinoma (CRC) is one of the most common cancers and liver is the predominant site of metastases and is the initial site in 30% of the distant metastases [1]. In unresectable, metastatic colorectal carcinoma, the first-line palliative chemotherapy consists of combination chemotherapy with 5- fluorouracil (5-FU), leucovorin (LV), and oxaliplatin (FOLFOX) and 5-FU/LV/irinotecan (FOLFIRI) [2]. Evaluation of the liver metastases after chemotherapy is important in order to guide subsequent treatment and to make possible more effective salvage treatment that prolongs the patient survival [3]. Currently, the revised Response Evaluation Criteria in Solid Tumors (RECIST) guidelines (version 1.1) are most widely used to assess the response to treatment for solid tumors, based on measurement of the longest diameter of the target lesions [4]. However, there are several problems in unidimensional measurement, such as the difficulty in determining the diameter of irregular and conglomerate masses, discrepancies in the scan planes leading to measurement error, and inter-observer variability [5, 6].

Quantification of the tumor burden using CT and MRI has become an issue in place of uni- or bidimensional measurement [7–9]. 3D measurement has advantages such as, more accurate assessment of tumor change, and superior measurement of an irregular mass [5]. With the recent advances in three-dimensional ultrasound (3D US) and its various clinical applications of volumetric measurement, oncologic measurement using 3D US has also been recommended [10–13]. Compared with CT or MR, US is more readily available and has no radiation hazard. Therefore, in cancer patients who require frequent follow-up examinations, 3D US can be a useful and successful method for monitoring their treatment response. However, many studies regarding volume measurement using 3D US were experimental or *in vitro* phantom studies due to the limited sonographic window using a 3D transducer and associated with varying patient anatomy and position as well as their respiratory motion [10, 12, 14–18].

With the technological revolution of 3D printing in the medical field, 3D visualization of human anatomy and variable pathologic conditions and the creation of 3D-printed physical models became accessible in the diagnostic practices [19–23]. Furthermore, 3D modeling technology can produce patient-tailored tumor models utilizing CT information. In this study, for the first time we developed 3D printing hepatic tumor models from patients’ CT data which are adequate for US evaluation. The purpose of this study is to evaluate the accuracy and reliability of 3D US for the evaluation of hepatic metastasis from CRC using 3D printing, patient-tailored tumor models obtained from CT images.

## Materials and methods

### Patient selection and study protocol

**Fig 1:**
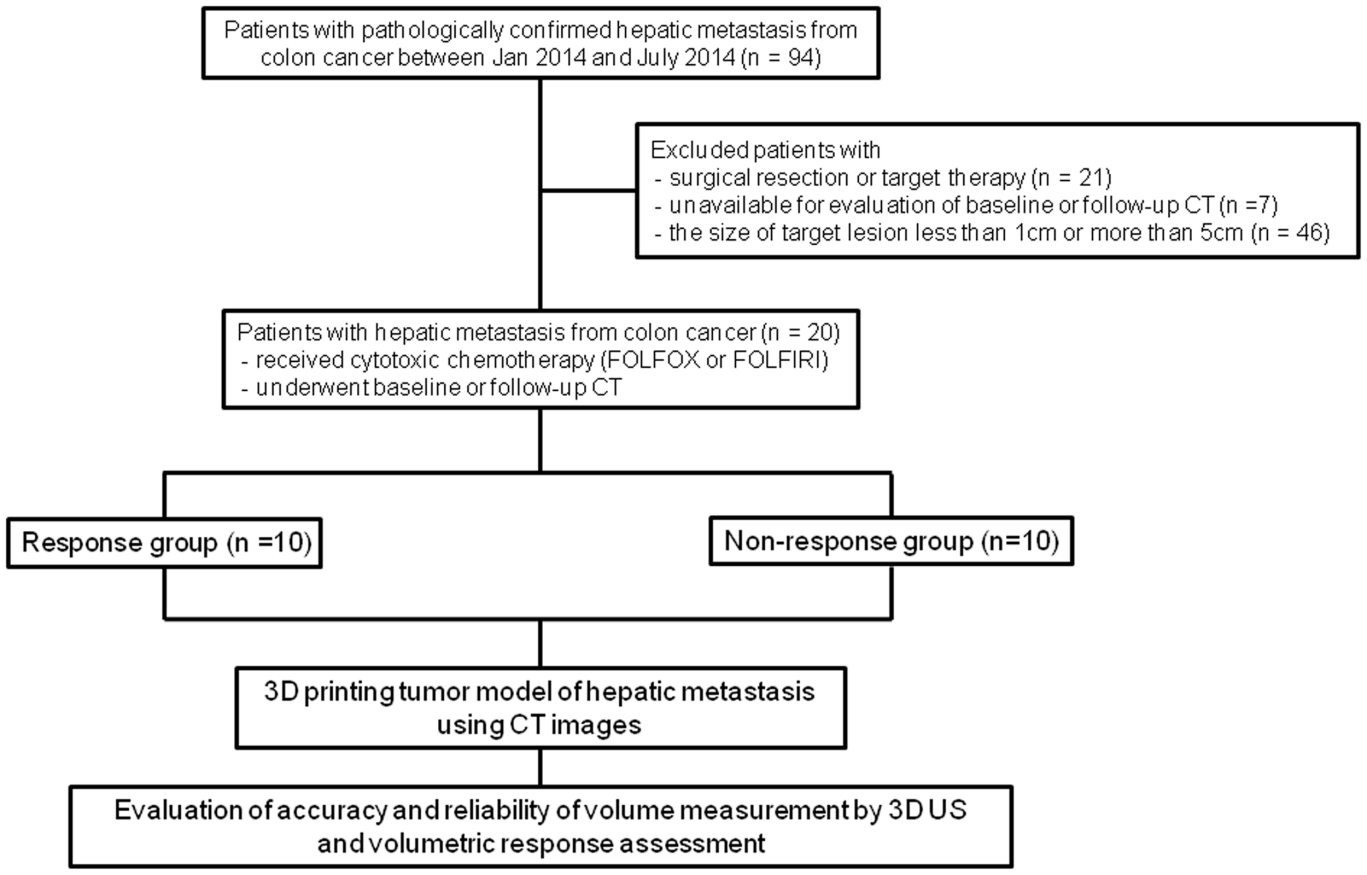
Study flowchart of patient selection and phantom construction.

This retrospective study was approved by our institutional review board in Seoul National University Hospital, and informed consent was waived. From January 2014 to July 2014, 94 patients were pathologically confirmed CRC with liver metastasis. The exclusion criteria included patients who underwent surgical resection or target therapy (n = 21), who were unavailable for evaluation of the baseline or follow-up CT (n = 7), and in whom the size of the target lesion was less than 1 cm or more than 5 cm (n = 46). Finally, 20 patients (17 men and three women, mean age, 58.4 years ± 9.5) who underwent cytotoxic chemotherapy including FOLFOX or FOLFIRI and who also underwent baseline and follow-up CT, were included in our study. Baseline demographic characteristics of patients with response group and non-response group are shown in S1 Table. Fig 1 shows a flowchart of the study population and the study protocol.

### CT examination

CT examinations were performed using the following CT scanners: Discovery CT750 HD (64-channel scanner, GE Healthcare, Milwaukee, WI, USA, n = 4), Brilliance 64 (64-channel scanner, Philips Healthcare, Cleveland, OH, USA, n = 10), Sensation 16 (16-channel scanner, Siemens Medical Solutions, Erlangen, Germany, n = 5), and LightSpeed Ultra (8-channel scanner, GE Healthcare, Milwaukee, WI, USA, n = 1). For 8-, 16-, and 64-detector CT examinations, detector collimations of 1.25, 0.75, and 0.625 mm, respectively, were used. A section thickness of 3.0–3.2 mm with a 2.5 to 3-mm reconstruction interval, a field of view of 300–370 mm, a gantry rotation time of 0.5 s, an effective amperage setting of 150–200 mAs, and a peak voltage of 120 kVp were used for all of the CT scanners. All patients underwent dual-phase CT during the arterial and portal venous phases. For dynamic phase imaging, a fixed dose of 1.5 ml of nonionic contrast material (iopromide [370 mg of iodine permilimeter], Ultravist 370; Schering, Berlin, Germany) per kilogram of body weight (555 mgI/kg) was injected at a rate of 2.0–4.0 mL/sec using a power injector (Multilevel CT; Medrad, Indianola, PA, USA).

### Response evaluation

The overall response was determined using the revised RECIST guidelines (version 1.1) [4]. Two radiologists (000, 000) evaluated in consensus the baseline CT and post-chemotherapy CT images after the fourth cycle. According to the RECIST guidelines, patients with complete response (CR) and partial response (PR) were categorized into the response group and patients with stable disease (SD) and progressive disease (PD) were categorized into the non-response group. Among the target tumor lesions in the patients of both the response and non-response groups, in order to construct a 3D printing phantom and obtain an appropriate acoustic window on 3D US, tumors less than 1 cm and more than 5 cm in diameter were excluded in this study. A total of 40 target lesions from each pre- and post-chemotherapy CT scan of 20 patients including 10 patients from response group and 10 patients from non-response were selected.

### 3D-printing hepatic tumor model

One radiologist (000) measured the regions of interest (ROI) of the representative target mass. PC-based, in-house software (MISSTA—medical imaging solution for segmentation and texture analysis) reconstructed the 3-dimensional, volume-rendering model and automatically calculated its volume using the input of ROI information. ROIs were delineated around the boundary of the tumor in each axial images. In order to minimize measurement errors, we used the mean value of three measurements obtained on different days. In-house software (MISSTA) was used for lesion segmentation with automated quantification of the tumor volume implemented using a dedicated C++ language with MFC (Microsoft Foundation Classes, Microsoft, Redmond, WA, USA).

**Fig 2:**
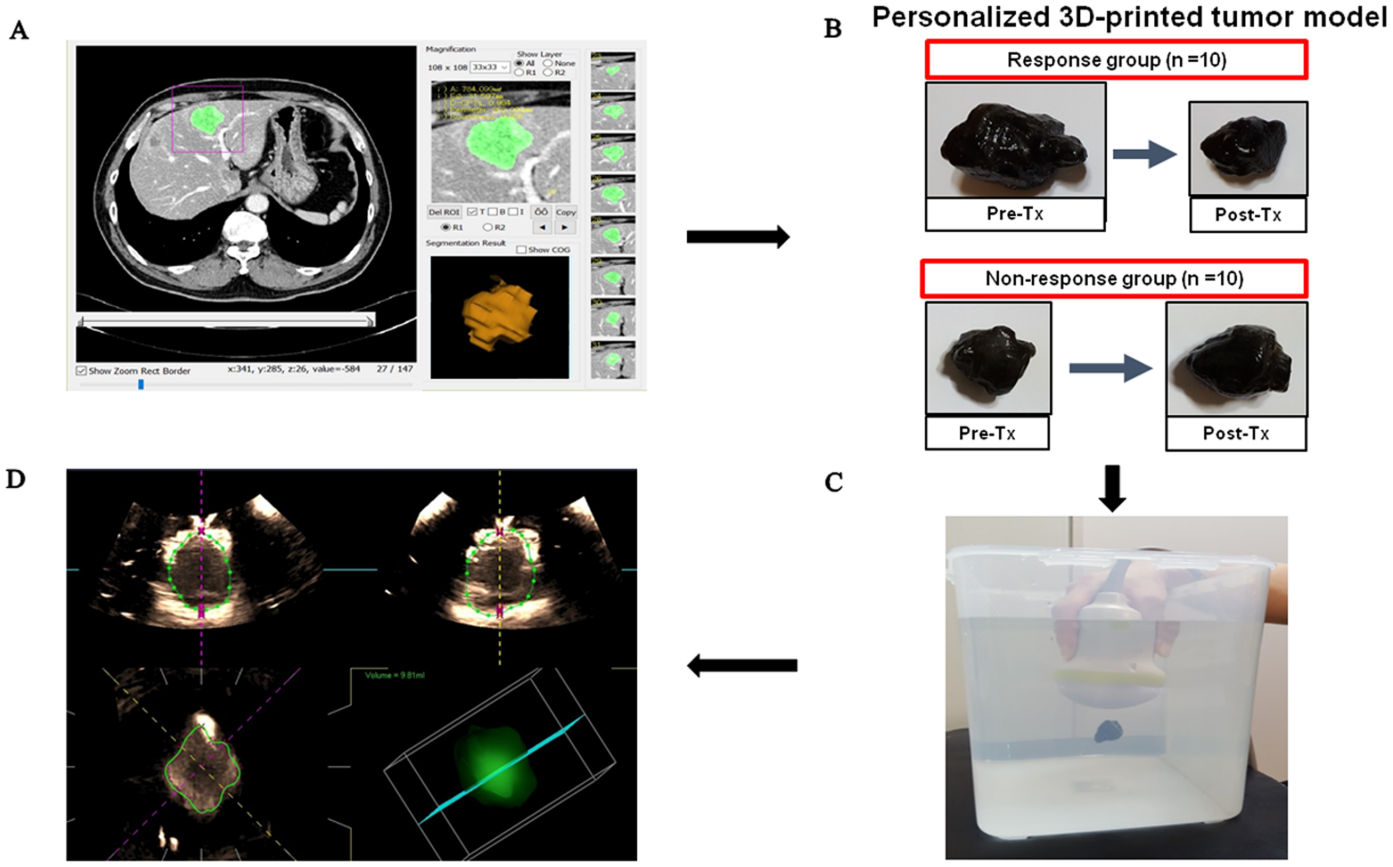
Study process flowsheet. **A**) Screenshot of the in-house program of segmentation and 3-dimensional volume-rendering reconstruction of the tumor. **B**) A personalized 3D-printed phantom tumor model constructed by the software and 3D printer. We had irereguler shaped 3D-printed phantom tumor model using the baseline CT and post-chemotherapy CT images in both response and non-response groups. **C**) Experimental setting for sonographic volume measurement of the phantom using 3D-transducer scanning through an automated sweeping movement. **D**) Volume measurement of the phantom. Manual outlining of the boundaries of the tumor phantom at 8 images of transverse (upper left) or longitudinal (upper right) plane. Then, boundaries at coronal plane (lower left) and 3D reconstructed image and its volume (lower right) were automatically generated by the built-in software of the ultrasound unit.

For fabricating a 3D-printing, hepatic tumor model, we initially segmented the tumor volume and made a phantom mold from the CT data, after which the volume files were converted to stereolithography files of mesh structures. The STL files were when transformed into a printable code format. A total of 40 tumor phantom molds of 20 patients at a 1:1 scale were produced using the MakerBot Replicator 2X 3D printer (Makerbot, New York, NY, US). After finishing these steps, the silicone material mixed with graphite powder for echogenicity was cast into the tumor phantom mold. Fig 2 shows a flowchart of the 3D-printing hepatic tumor model.

### Tumor volume measurement using 3D-US

Two radiologists (000, 000) independently performed the scanning using an US unit (Aplio 500; Toshiba Medical, Otawara, Japan) equipped with PVT-375BT, a 3.5 MHz curved 2D-transducer, and a PVT-375MV, 3.5 MHz mechanical convex 3D-transducer with the following parameters: a dynamic range of 65; a gain of 85; a frame rate of 25 fps; and a depth of 10 cm in a 2D-transducer, a dynamic range of 65; a gain of 89; a frame rate of 30 fps; and a depth of 9 cm in a 3D-transducer. After each phantom was fixed with a fine thread in the center of a container filled with distilled water, the volume transducers were dipped in the water and placed over 2 cm above the phantom. On 2D US, the phantom was imaged using the maximum transverse plane. The radiologist then adjusted the size and position of the volume of interest (VOI) so as to contain the phantom. During an automated sweeping movement through a predetermined sweeping angle of 75°, the radiologist held the transducer in order to avoid any movement. Volumetric measurement of each phantom was performed on the US unit using the analysis software. The software allowed simultaneous display on the monitor in three, different, 2D perpendicular planes. One 2D plane was selected, and a rotation axis that passed through the center of the tumor to be measured was set on the 2D image. The outer boundary of a tumor was manually drawn on the 2D image, after which the volume data were rotated on the rotation axis by 22.5° in order to produce the next 2D image. Because each rotation step was 22.5°, each measurement required eight rotation steps, manual drawing of the boundaries on 2D images was performed a total of eight times (Fig 2). In addition to the tumor volume measurement on US, we estimated the volume of the phantom using the ellipsoid volume formula, $\text{V=}\frac{4}{3}\pi \;r_{1}r_{2}r_{3}$, where *r*_1_, *r*_2_ and *r*_3_ are half the diameters on each of the x, y, z planes as seen on 2D US. The reference volume of the phantom was automatically calculated and indicated on the PC-based, in-house software (MISSTA) that was used for modelling 3D phantoms with CT images. The actual volume of 3D-printed phantoms was measured using the water displacement method and compared with the reference volume of CT images.

### Statistical analysis

In order to evaluate the accuracy of the volume measurement, the mean difference and standard deviation were calculated. The limits of agreement and 95% confidence intervals were determined using the methods described in a publication by Bland and Altman [24]. The inter-observer reliability was evaluated using the intraclass correlation coefficient (ICC) and the limits of agreement. An ICC > 0.7 was considered to indicate an excellent correlation. Comparison of the 3D US volume analysis and the RECIST guidelines for determining the patient response to treatment was performed using kappa statistics. The kappa value of the inter-observer agreement was assigned as follows: less than 0.20, poor; 0.21–0.40, fair; 0.41–0.60, moderate; 0.61–0.80, good; and more than 0.81, excellent. A *p* value less than 0.05 was considered to indicate a statistically significant difference. Statistical analyses were performed using the Statistical Package for Social Science (SPSS version 19.0 for Microsoft Windows) and Medcalc (version 16.2.1, Medcalc Software, Mariakerke, Belgium) statistical software.

## Results

The lesion diameters ranged from 10.6 to 33.8 mm (mean 21.8 ± 6.5 mm). In the response group, the mean diameter decreased from 26.9 ± 5.3 mm to 16.1 ± 3.2 mm after chemotherapy. In the non-response group, the mean diameter changed from 21.5 ± 6.7 mm to 22.8 ± 5.9 mm after chemotherapy. There was no technical failure in creating the personalized 3D printed tumor model.

### Accuracy and reliability of volumetric US

**Fig 3:**
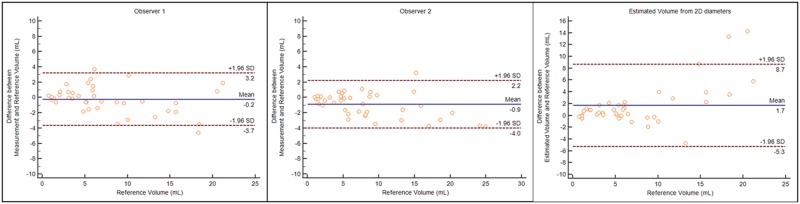
Comparison of measured volume using three-dimensional ultrasound and estimated volume from 2D diameters $\left(\text{V=}\frac{\pi }{6}abc\right)$ with the true volume of tumor phantoms. **A-C**) Plots of difference between the volume measurement and estimation against the true volume. The 95% limits of agreement (mean difference ± 1.96 SD) calculated using the Bland and Altman method were indicated as dashed line.

The reference volume from CT images was 7.42 ± 5.76 mL (mean ± SD) and the volume of 3D printed phantoms using the water displacement method was 7.44 ± 5.80 mL (mean ± SD) without significant difference (p>0.05). The tumor volume measurement by observers 1 and 2 using 3D US were 7.18 ± 5.44 mL and 8.31 ± 6.32 mL, respectively, without significant difference from the tumor volume from CT (*p*>0.05). The tumor volume measurement on 3D US showed better correlation with CT tumor volume than the estimated tumor volume on 2D US, without a significant difference. The correlation coefficients (r) were 0.953, 0.97, and 0.945 for 3D US measurement by observers 1 and 2, and the estimated volume. In addition, the mean difference and the limits of agreement were less in the measured volume than in the estimated volume (Fig 3). Table 1 summarizes the comparison of the measured volume using 3D US and the estimated volume from 2D US with CT tumor volume. Regarding the reliability of the volume measurement on 3D US, an excellent reliability correlation was observed (ICC = 0.978, 95% CI, 0.958–0.988).

**Table 1:** Comparison of measured volume using three-dimensional ultrasound and estimated volume from 2D diameters $\left(\text{V=}\frac{4}{3}\pi r_{1}r_{2}r_{3}\right)$ with the true volume of tumor phantoms.

	Measured Volume (observer 1)	Measured Volume (observer 2)	Estimated volume from 2D diameters $\left(\text{V=}\frac{4}{3}\pi r_{1}r_{2}r_{3}\right)$
**Mean volume**^a^	7.18 ± 5.44	8.31 ± 6.32	9.10 ± 8.47
**Mean difference from reference volume (95% CI)**	-0.24 (-0.79 to 0.32)	0.89 (0.38 to 1.40)	1.69 (0.55 to 2.82)
**SD of differences between measured volume and reference volume**	1.75	1.58	3.56
**Upper limit of agreement**	3.19 (2.23 to 4.15)	3.99 (3.12 to 4.86)	8.66 (6.70 to 10.62)
**Lower limit of agreement**	-3.66 (-4.63 to -2.70)	-2.21 (-3.08 to 1.34)	-5.29 (-7.25 to 3.33)

^a^ The mean reference volume was **7.42 ± 5.76 mL**

### Treatment response evaluation

Table 2 summarizes the changes in diameters of the tumor on CT and 3D US, and corresponding response evaluation according to the unidimensional and volumetric RECIST criteria. The average change in the unidimensional diameter on pre- and post-chemotherapy CT was 33.27%, whereas the percentage change in CT volume was 64.0%. In the response group, an average of a 43.49% was decreased in diameter, whereas a 71.42% was decreased in volume. In the non-response group, an average of a 10.15% was increased in diameter, while there was a 22.33% increase in volume.

**Table 2:** Changes in pre- and post-chemotherapy diameters of the target lesion on CT and volumes of the phantoms on 3D US, and corresponding response evaluation according to the unidimensional (1D) RECIST criteria and three-dimensional (3D) volumetric criteria.

No.	Unidimensional RECIST Criteria*				Volumetric Criteria**											
					CT volume				3D US volumetric US (R1)				3D US volumetric US (R2)			
	Baseline (cm)	Post-Tx. (cm)	Size Change	Criteria	Baseline (mL)	Post-Tx. (mL)	Volume Change	Criteria	Baseline (mL)	Post-Tx. (mL)	Volume Change	Criteria	Baseline (mL)	Post-Tx. (mL)	Volume Change	Criteria
**1**	1.8	1	44.5% D	PR	5.15	1.06	79.4% D	PR	4.63	0.8	82.7% D	PR	4.38	1.27	71.0% D	PR
**2**	3.3	1.8	45.5% D	PR	15.65	3.12	80.1% D	PR	14.94	3.1	79.3% D	PR	18.57	3.1	83.3% D	PR
**3**	2.2	1.5	31.8% D	PR	6.46	2.86	55.8% D	**SD**	5.13	4.63	9.7% D	SD	8.32	5.72	31.3% D	SD
**4**	4	1.5	62.5% D	PR	20.5	2.0	90.2% D	PR	21.33	2.32	89.1% D	PR	24.14	1.88	22.1% D	PR
**5**	3.3	2	39.4% D	PR	18.27	4.24	76.8% D	PR	13.7	4.45	67.5% D	PR	20.25	4.62	77.2% D	PR
**6**	3.4	2.3	32.4% D	PR	18.4	5.92	67.8% D	PR	14.89	7.42	50.2% D	**SD**	15.16	5.97	60.6% D	**SD**
**7**	2.4	1.4	41.7% D	PR	5.41	1.31	75.7% D	PR	3.91	1.3	66.8% D	PR	4.83	1.51	68.7% D	PR
**8**	2.7	1.4	48.1% D	PR	6.04	1.61	73.4% D	PR	6.22	0.98	84.2% D	PR	5.12	1.43	72.1% D	PR
**9**	3	2.1	30% D	PR	8.74	4.79	45.2% D	**SD**	8.12	2.98	63.3% D	SD	7.62	4.65	39.0% D	SD
**10**	3.9	1.6	59.0% D	PR	10.14	3.06	69.8% D	PR	13.07	3.78	71.1% D	PR	13.12	3.73	71.6% D	PR
**11**	2.9	2.4	17.2% D	SD	10.02	5.04	49.7% D	SD	7.12	4.41	38.1% D	SD	9.72	5.13	47.2% D	SD
**12**	2.8	3.3	17.9% I	SD	11.74	14.75	25.7% I	SD	11.25	12.98	15.4% I	SD	13.33	15.84	18.8% I	SD
**13**	3.3	2.8	15.2% D	SD	15.66	21.16	35.1% I	SD	13.81	23.1	67.3% I	SD	14.86	24.93	67.8% I	SD
**14**	1.2	1.8	50% I	PD	1.09	2.06	89.7% I	PD	0.91	2.82	209.9% I	PD	1.02	2.43	138.2% I	PD
**15**	2.8	2.0	28.6% D	SD	9.53	3.57	62.6% D	SD	8.82	4.19	52.5% D	SD	8.82	5.24	40.6% D	SD
**16**	2.4	2.4	0%	SD	5.69	5.39	5.2% D	SD	8.14	7.34	9.8% D	SD	8.07	7.18	11.0% D	SD
**17**	1.6	1.8	12.5% I	SD	6.09	5.87	3.6% D	SD	9.79	6.83	30.2% D	SD	9.5	8.1	14.7% D	SD
**18**	1.1	1.2	9.1% I	SD	0.72	1.09	50.1% D	SD	1	1.16	16% I	SD	1.54	2.1	36.4% I	SD
**19**	1.6	2.7	68.8% I	PD	3.5	8.85	153.2% I	PD	3.38	5.4	59.8% I	**SD**	5.69	8.7	52.9% I	**SD**
**20**	2.6	2.8	7.7% I	SD	6.94	13.24	90.8% I	**PD**	6.45	10.7	65.9% I	**SD**	7.77	16.96	118.3% I	PD

*1D RECIST criteria: PR- at least 30% decrease; PD- at least 20% increase; SD- between 30% decrease and 20% increase in diameter

**3D criteria: PR- at least 65% decrease; PD- at least 73% increase; SD- between 65% decrease and 73% increase in volume

D: decrease, I: increase

For therapeutic response assessment using volumetric measurement, unidimensional RECIST criteria was extrapolated to volume criteria. We used the formula $\text{V=}\frac{4}{3}\pi \;r^{3}$ where r is half the diameters for extrapolation of unidimensional criteria to the volumetric criteria. Therefore, PR represented a greater than 65% reduction in volume (1–0.7^3^ ≅ 0.65), PD represented a greater than 73% increase in tumor volume (1.2^3^ ≅ 1.73), and SD indicated less than a 65% reduction to less than a 73% increase in tumor volume [25]. After applying the volumetric criteria, two patients with PR in the response group changed to SD, and one patient with SD in the no-response group changed to PD based on the CT volume. Overall, disagreement in three patients according to the RECIST criteria and change to the no-response group in two patients were observed after volumetric response evaluation. Compared to the response evaluation based on the CT volume, the response evaluation based on 3D US was concordant in 17 out of 20 patients for observer 1 and in 18 out of 20 patients for observer 2. In terms of the response versus the non-response group, the CT volume and the 3D US volume measurement were identical in 19 of the 20 patients for both observers with excellent inter-observer agreement (κ = 0.961).

## Discussion

We found that using CT tumor volume as a reference standard, volumetric 3D US with a personalized 3D-printed tumor model from CT showed no statistically difference (7.18 ± 5.44 mL for observer 1 and 8.31 ± 6.32 mL for observer 2 vs 7.42 ± 5.76 mL on CT, p>0.05). In addition, 3D US provided a high correlation coefficient with CT volume (r = 0.953, observer 1; r = 0.97, observer 2) and a high inter-observer reliability (ICC = 0.978, 0.958–0.988). Regarding the response assessment, the percentage change in CT volume was greater than the percentage change in diameter on both pre- and post-chemotherapy (64.0% vs 33.27%). 3D US was in agreement with the CT volume in 17 of the 20 patients for observer 1 and in 18 of the 20 patients for observer 2 and with excellent inter-observer agreement (κ = 0.961).

Previous studies regarding the volumetric 3D US used manually made phantoms, such as pieces of ham, or tissue phantoms made using pieces of chicken or pork [10, 16, 17]. In this study, we first reconstructed the hepatic tumor applying a personalized 3D-printed tumor model from CT images. Our study results showed that 3D US has no statistically significant difference compared to CT tumor volume, and thus providing a high value of correlation coefficient as well as high inter-reader agreement. These results are concordant with the results of previous studies [10, 16, 17, 26, 27]. The absolute measurement error in Park’s study was 2.6 mL ± 0.2 mL (mean ± SD) using an US phantom made of ham with high agreement between observers [10]. Xu et al. demonstrated that the volume measurement error of 3D US was 0.3% ± 3.3% in regular phantoms, -0.4% ± 3.7% in irregular phantoms, and 0.9% ± 11.3% in liver tumor compared with -5.3 ± 9.4%, 13.6 ± 28.0%, and 15.3 ± 37.3%, respectively, for 2D US [16]. In their *in vivo* study conducted using 68 liver tumors, 31 liver tumors was unsuccessfully measured for various reasons including tumor rupture or bleeding, and inability to separate a tumor from liver tissue. In contrast, using 3D-printing technology, we could reconstruct personalized hepatic tumor phantoms using each patient’s CT data and, therefore, ascertain the true volume of a tumor without surgical resection. Furthermore, we could also evaluate the change in the volume of a hepatic tumor after treatment. With the technological revolution of 3D printing in the medical field, 3D visualization of anatomy and pathologic conditions and creation of 3D-printed physical models became accessible in the diagnostic imaging practices [19–23]. In many cases, the 3D modeling has been used for patients with complex disease or anatomy in the preoperative setting. Recently, personalized or realistic experimental phantoms were constructed for validation of new imaging techniques [28] [29]. In our study, we reconstructed hepatic tumor models utilizing CT information to validate the 3D US quality and to investigate the volumetric criteria in the chemotherapy patients. Therefore, compared with the other phantom studies regarding 3D US, our patient-tailored, hepatic tumor models can simulate the real tumor morphology and changes according to the treatment.

Although RECIST criteria are most widely used to assess the response to treatment for solid tumors, its limitations associated with unidimensional measurement, such as difficulty in determining the diameter of irregular or conglomerated lesions, have been a dilemma in the clinical fields [5, 6]. Instead, volumetric response evaluation has gained much interest and acceptance in place of uni- or bi-dimensional methods [7–9, 30, 31]. Volumetric measurement has advantages regarding better quantification of the total tumor burden, more accurate assessment of tumor change, and better measurement of irregular masses [5]. However, there have been only a few studies regarding the accuracy and reliability of volumetric response evaluation, and there are currently no established volumetric criteria. In our study, compared to the RECIST criteria, volumetric criteria based on the CT made a change in the response evaluation in 15% (3/20) of the patients (PR to SD in two patients, SD to PD in one patient) and a change in the group in 10% (2/20) of the patients, i.e. response group to no-response group. Our discordance rate of 15% was similar to previous studies [3, 5]. Fang et al. demonstrated that volumetric evaluation showed good agreement with RECIST (κ = 0.779) and the discordance rate was 13.3% (6/45) [3]. In our study, two patients with PR determined by RECIST were considered as SD by volumetric assessment. This could be explained in that although a larger change in volume than in longitudinal diameter was observed in the study, the volumetric criteria derived from extrapolation of unidimensional criteria had a much wider range for stable disease, i.e. 65% reduction to 73% increase. Therefore, a new, validated, volumetric guideline is needed rather than simple transformation using the volumetric formula of the RECIST unidimensional criteria.

With the advances in 3D US technology, it has been reported that 3D US is accurate and reliable for volume measurement in various fields [10, 32–35]. However, with regard to clinical application, 3D US techniques are more difficult for the user to manipulate than conventional US techniques due to bigger assemblies and longer time spending on the data acquisition and image interpretation process [12]. Therefore, many studies have been experimental or *in vitro* phantom studies because of the limited sonographic window using a 3D transducer [10, 12, 14–18]. Furthermore, small US window of intercostal and substernal view for the hepatic tumors in the human subjects makes more difficult to cover the whole target lesion with a big 3D transducer. Due to such limitations of 3D US for application in hepatic metastasis of CRC patients, we reconstructed the patients’ hepatic tumors using 3D printing method, and validated the accuracy and reliability of 3D US measurement. Therefore, future improvement of scanning conditions of 3D US, including development of smaller probe for better sonic window, improvement of scanning speed, and simplification of manipulating method, will enable us to use 3D US in the regular follow-up examinations after chemotherapy in the metastatic CRC patients. Furthermore, if new volumetric criteria are established in the future, we can use 3D US as a follow-up tool in place of or complementary to CT for assessment of volumetric treatment response in the patients undergoing chemotherapy.

Our study has several limitations. First, as we did not fully understand the acoustic characteristics of the 3D-printed phantoms made up of silicone and graphite powder, measurement error owing to the thick echogenicity at the interface between the phantom and water was inevitable. Despite the high inter-observer agreement (ICC = 0.978) in 3D US measurement, different individual measurement tendencies were observed. For optimal visualization of phantoms on 3D US, further studies with in-depth knowledge of the acoustic characteristics of the various materials used for 3D printing will be needed. Second, we did not evaluate intra-observer variability, i.e. the variability of repeated volume measurement by the same observer. The factors influencing measurement variability including depth of field of the image and segmentation of the tumor in each plane are more affected by different observers, thus inter-observer variability during regular follow-up US exams are more important issue in the clinical field rather than intra-observer variability. However, further study with evaluation of intra-observer variability would be needed to validate the reliability of 3D US volumetry. Third, although we simulated hepatic metastasis of CRC patients using 3D printing phantoms for evaluation of 3D US volumetric measurement, for clinical application of 3D US we need further study for validation of this technique in the patients with hepatic metastasis.

In conclusion, 3D US volumetric measurements applying a personalized, 3D-printed tumor model using CT in patients with hepatic metastasis from CRC, constitute an accurate and reliable method for the response evaluation compared with the tumor volume from CT. With the advantages of accessibility, high cost-effectiveness, and no radiation hazard compared with CT and MRI, 3D US would be useful in the volumetric treatment response evaluation of cancer patients.

## Supporting information

S1 Table Baseline demographic characteristics of the study populations. (PDF)

## Abbreviations

3D US: three-dimensional ultrasound
3D US: three-dimensional ultrasound
CR: complete response
CRC: colorectal carcinoma
FOLFIRI: 5- fluorouracil, leucovorin, and irinotecan
FOLFOX: 5- fluorouracil, leucovorin, and oxaliplatin
ICC: intraclass correlation coefficient
PD: progressive disease
PR: partial response
RECIST: Response Evaluation Criteria in Solid Tumors
ROI: regions of interest
SD: stable disease
