# Supplementary material for: Therapeutic response assessment using 3D ultrasound for hepatic metastasis from colorectal cancer: Application of a personalized, 3D-printed tumor model using CT images
Source: PLoS One. 2017 Aug 10;12(8):e0182596. doi: 10.1371/journal.pone.0182596 (PMC5552302; doi:10.1371/journal.pone.0182596)
Supplement: S1 Table — (PDF) [file pone.0182596.s001.pdf]

S1 Table. Baseline demographic characteristics of the study populations

Characteristics		Response group (N =10)	Non-response group (N =10)	P value
**Sex**	Male<br>Female	7<br>3	10<br>0	0.067
**Age**	Median (years)<br>Range	52.5<br>46-75	60.0<br>41-71	0.782
**Localization of primary tumor**	Colon<br>Rectal	9<br>1	6<br>4	0.131
**T-stage**	T1/T2<br>T3/T4<br>NA	2<br>7<br>1	2<br>8<br>–	0.587
**N-stage**	N1<br>N2<br>NA	5<br>4<br>1	5<br>5<br>–	0.574
**Resection of primary tumor**	No<br>Yes	1<br>9	–<br>10	0.317
**Localization of metastasis**	Liver<br>Liver only<br>Lung<br>Peritoneal	10<br>9<br>–<br>1	10<br>6<br>1<br>1	0.463
**No. of metastatic sites**	1<br>≥2	9<br>1	6<br>4	0.131
**CEA level at baseline**	<200 $ngml^{-1}$<br>≥200 $ngml^{-1}$	9<br>1	10<br>–	0.317
**Chemotherapy agents**	FOLFOX<br>FOLFIRI	8<br>2	7<br>3	0.615
